# Oral Health Attitudes among Preclinical and Clinical Dental Students: A Pilot Study and Self-Assessment in an Egyptian State-Funded University

**DOI:** 10.3390/ijerph18010234

**Published:** 2020-12-30

**Authors:** Mohamed Mekhemar, Kamal Ebeid, Sameh Attia, Christof Dörfer, Jonas Conrad

**Affiliations:** 1 Clinic for Conservative Dentistry and Periodontology, School of Dental Medicine, Christian-Albrecht’s University, 24105 Kiel, Germany; doerfer@konspar.uni-kiel.de (C.D.); conrad@konspar.uni-kiel.de (J.C.); 2 Fixed Prosthodontics Department, Ain Shams University, Cairo 11566, Egypt; kamal_ebeid@dent.asu.edu.eg; 3 Department of Cranio-Maxillofacial Surgery, Justus-Liebig University Giessen, Klinikstrasse 33, 35392 Giessen, Germany; Sameh.Attia@dentist.med.uni-giessen.de

**Keywords:** oral health attitudes, dental students, Germany, oral hygiene, Egypt, Ain Shams University, preclinical–clinical transition

## Abstract

Dentists should present to patients as good role models in their oral health behaviour. Previous studies have demonstrated how education can improve dental students’ oral health. This pilot investigation aimed to compare and evaluate the features of the oral health behaviour and attitudes of preclinical and clinical dental students at Ain Shams University, a public Egyptian university. The Hiroshima University-Dental Behaviour Inventory (HU-DBI) survey was provided to 149 (78 female/71 male) dental students. Dichotomised (agree/disagree) answers to 20 HU-DBI items were possible, with a maximum conceivable score of 19. An estimation of oral health behaviour and attitudes was calculated by the sum of correct oral health answers to every item by the study groups and evaluated statistically. The score of oral health-favouring answers was higher in clinical (11.50) than preclinical students (10.63) and was statistically significant (*p* < 0.05). Single-item evaluations showed no statistical significance, except in one survey item. This survey exhibited weak differences in the improvement of oral hygiene behaviour and attitudes between participating preclinical and clinical students, as well as overall poor oral health behaviour in both groups. This inadequacy of Egyptian public dental education in terms of sufficient student oral health progress emphasises the necessity for supplementary courses and curricular reviews that accentuate the need for future dentists to display the correct oral health behaviour.

## 1. Introduction

Diseases and disorders of the oral cavity are amid the most pervasive conditions worldwide and produce abundant burdens in relation to wellbeing and economic difficulties, significantly decreasing the quality of life of those living in the affected countries and societies [1]. Education regarding the correct health behaviour is reported to be an essential component for the successful prevention of diseases in numerous medical fields, such as dentistry and oral public health [2]. Being one of the most effective approaches to educate the public about oral health, the self-applied oral hygiene measures and related behaviour and attitudes of dentists can immensely impact a society’s oral health [3]. These measures exhibit the self-perceived preference of oral healthcare professionals toward oral health and are translated practically through their oral health behaviour as they aim to become decent role models of the mindset and habits required for adequate oral health. Therefore, maintaining good attitudes and behaviour toward self-applied oral hygiene measures is one of the most important tasks of dentists in any society [3].

Egypt is considered one of the most inhabited countries in the Middle East and Africa, with a population of accelerating growth that is expected to reach 120 million by 2030 [4]. Most of Egypt’s citizens live in urban regions, mostly within the densely populated mega-centres of Cairo and Alexandria, besides the other major cities of the Nile Delta and along the Suez Canal [5]. These areas are considered amongst the world’s most densely populated locations, with more than 1540 persons per square kilometre, where health-related quality of life and oral public health have been demonstrated to be the main problems [5,6,7,8]. The Egyptian education system, one of the largest educational structures in Africa and the Middle East, involves a highly extensive network of higher education organisations, with 25 public universities and nearly 20 private educational institutions [9]. Medical and dental education and training in Egypt was initiated in 1827 and became part of university courses in 1919, making Egypt a pioneer in the field of medical education in its geographic location [9,10]. Up to the present time, most dental schools in Egypt have followed the French educational structure, embracing a five-year programme of undergraduate dental education, as well as a one-year internship before obtaining a dental practicing license [9]. Starting with a three-year preclinical period, the student obtains mostly theoretical information about oral hygiene and healthcare, with minimal applied or practical training on artificial teeth and dummy heads. This educational phase mostly focuses on physics, natural sciences, dental materials, laboratory dentistry, and different branches of general medicine. Following this stage, students start two clinical years in which they attend lectures and perform clinical treatment on real patients. Subsequently, a Bachelor of Medicine and Surgery degree is awarded to the student upon successful examinations and graduation. Finally, graduates must also attend an additional one-year internship programme in different dental departments and specialties in order to obtain authorisation to work as a general dental practitioner [9].

This Egyptian dental education model defines a strong preclinical–clinical contrast and uses English as the teaching and examination language. Furthermore, it implements discipline-based curricula, in which large-group educational lectures and apprenticeship methods of clinical training are the primary methods of teaching [9]. This sharp transition from the preclinical to the clinical phases of education has profound effects on dental students, as they shift from their function as recipients of theoretical oral hygiene education to becoming contributors and educators themselves in charge of actual patients’ oral health [11]. The literature underlines the need to revise the curricula to reinforce the professional skills of dental and medical students throughout this difficult phase of educational transition [11]. This revision has to permit an integration of theory and practice by clinical students, driving them to display the correct attitudes and to apply professionalism in regard to their own oral health behaviour as instructors and role models to their patients [3,12,13]. Lately, numerous reports have assessed this preclinical–clinical shift in diverse areas around the world by associating the oral health attitudes and behaviour of preclinical and clinical dental students as some of the first indications of the success of the educational transition. Several studies have shown that there is an obvious improvement in the oral health behaviour and attitudes in clinical students [12,13,14,15,16,17,18,19,20,21,22], while other studies have described a weaker preclinical–clinical adaptation [3,23,24]. When examining the available studies, it is clear that no comparable examination has been performed in an Egyptian state-funded university. Therefore, this pilot study aimed to evaluate the self-reported oral health behaviour and attitudes of preclinical and clinical dental students at Ain Shams University of Cairo, Egypt, using a modified Hiroshima University-Dental Behaviour Inventory (HU-DBI) survey. This study intended to provide an insight into students’ educational transformation and preparation to enter the clinical phase and to accomplish their role as oral health instructors.

## 2. Materials and Methods

### 2.1. Study Population and Methodology

**Figure 1:**
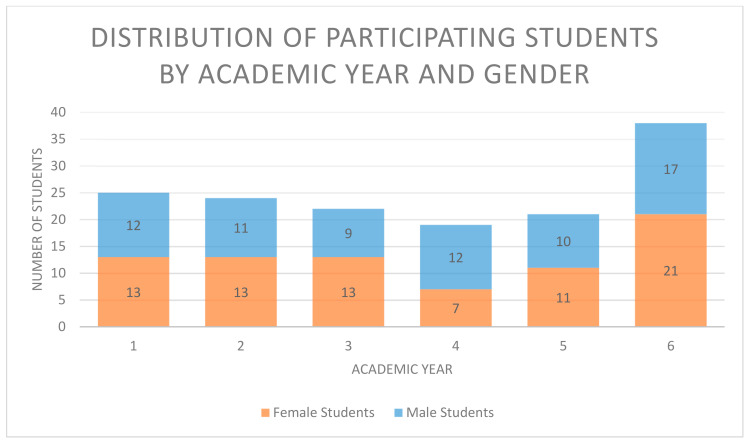
Total number of participating students by academic year and gender.

The HU-DBI is a well-established survey tool to inspect dental health perceptions and oral health-related attitudes and behaviour. It was previously translated from the Japanese original version into English and several other languages for diverse multicultural assessments [3,22,25]. The outcomes of these translations displayed the good validity and reliability of the survey and provided a solid foundation for similar investigations worldwide [3,21]. In the current investigation, oral health attitude and behaviour were evaluated via a shortened form of the HU-DBI English questionnaire [3,19,26], composed of 20 items associated with features of oral health attitudes and behaviour. Each item involved a dichotomous selection for its response (agree/disagree), as presented in previous surveys [3] (Table 1). After receiving ethical approval by the universities of Kiel (AZ D 431/17) and Ain Shams (D445/11), we conducted a pilot survey after morning lectures and clinical sessions for participation by dental students of all academic years within the dental faculty of Ain Shams University in Cairo, Egypt. The opening text of the survey briefly clarified the pilot study and guaranteed voluntary participation and anonymity. Encouragements or gifts were not given to the participants, and no personal data were collected, excluding academic year and gender. Suitable participants were dental students enrolled in courses in all academic years and no exclusion criteria in terms of age, gender, or nationality were defined (Table 2 and Table 3 and Figure 1).

### 2.2. Calculation of Sample Size

To estimate the number of participants required for a significant sample size in the pilot study, we defined a confidence level of 95% and a probability of 5% for the calculation, as presented previously in similar surveys and as described for sample size calculations in pilot studies [3,27]. According to these settings, we decided that, within this pilot study, at least 59 student participants were required for a statistically significant sample size.

### 2.3. Scores and Statistical Analysis of the Hiroshima University-Dental Behavioural Inventory

The HU-DBI delivers a measurable evaluation of candidates’ attitudes in relation to oral and dental health in correlation with the sum of agree/disagree answers given by the participants [3,23,28]. The overall oral health behaviour and attitudes of the participants were assessed by the calculation of the total score of their dichotomous replies (agree or disagree) to the single items of the questionnaire, as defined previously [3], counting one point for each response favouring good oral and dental health (Table 1). Similar to previous investigations [3], in the current survey of 20 items, the maximum conceivable score consists of 19 points, since the first item only labelled sociodemographic data. Higher points characterise improved attitudes and behaviour in relation to oral health. The Kolmogorov–Smirnov test was used to assess data normality, showing that the data were not normally distributed. Alterations between preclinical (1–3) and clinical (4–6) academic years, in addition to male and female students’ sum points, were evaluated by the Wilcoxon signed-rank test. Moreover, each item was examined separately by the Wilcoxon signed-rank test for a comparison of the correct and incorrect (“agree” and “disagree”) responses of preclinical and clinical students to this element of the questionnaire [3,23]. Data analysis was completed using SPSS software (IMB Corp., IBM SPSS Statistics, Version 27.0, Armonk, NY, USA). The significance level (*p*-value) for all tests was set at 0.05.

## 3. Results

A total of 149 students participated in the assessment, providing a statistically significant sample size for the pilot study. The contributors included 71 (48%) preclinical (academic years 1–3) and 78 (52%) clinical (academic years 4–7) students. Out of the total 149 students across all academic years, 25 volunteered to participate in the study from year one, 24 from year two, 22 from year 3, 19 from year 4, 21 from year 5, and 38 from year 6. Approximately 52% of the students were females and 48% males (Table 2 and Table 3). Almost 80% of the preclinical and clinical students were still living with their families during their university education. The percentage distribution of the students’ answers supporting or contradicting correct oral health, as explained above, is displayed in (Table 4).

### 3.1. Oral Health Attitudes

Preclinical and clinical groups showed a rate of nearly 70% for previous visits to the dentist. Almost 50% of both groups also visited a dental practice only for emergency treatments or when in pain. Nevertheless, the clinical dental students presented improved oral health compared to the preclinical group in all items related to oral health attitudes. The number of preclinical students who were bothered by the colour of their gingiva was significantly higher (62%) than their counterparts (43%) (*p* < 0.05) (Table 4).

### 3.2. Oral Health Behaviour and Self-Reported Oral Health

The clinical participants reported better oral health behaviour in almost all items of the survey. However, preclinical students showed better results regarding self-reported oral health behaviour in some aspects of the survey, as they used mouthwashes more regularly (49.3%), worried more about their tooth colour (71.8%) and mouth odour (74.6%), and reported that they snacked less on sweets during the day (69.0%) than the clinical participants (43.6%, 65.4%, 73.1%, and 74.4%, respectively) (Table 4).

### 3.3. Mean HU-DBI Scores and Statistical Significance

The general HU-DBI mean score of the answers favouring good oral hygiene was slightly higher in the clinical (11.50 ± 3.25) than preclinical (10.63 ± 2.64) students and showed statistical significance (*p* < 0.05) (Table 5).

The analysis of each item of the questionnaire exhibited no statistically significant differences between preclinical and clinical students, except for Item 15 of the survey (*p* < 0.05) (Table 4). When comparing the students’ responses by gender, we found that females demonstrated a significantly higher HU-DBI mean score (11.76 ± 2.79) than male participants (10.35 ± 3.06) (*p* < 0.05) (Table 5).

## 4. Discussion

In Egypt, as in most developing nations, diseases of the oral cavity are among the most prevalent conditions affecting public health [24]. Recently, several oral health investigations have been performed to detect and evaluate the incidence and origins of oral disease in Egyptian society [25,26,27]. To date, no study has evaluated the oral health attitudes and behaviour of dental students in Egyptian state-funded/public universities as future experts of oral health. Due to this deficit, this pilot investigation was conducted to evaluate and compare several facets of the oral health attitudes and behaviour between preclinical and clinical dental students in an Egyptian state-funded university. The main aim was to assess the outcome of the educational preclinical–clinical shift of the students and their oral health awareness as future health professionals and educators of the Egyptian population. Although a similar investigation was performed recently in one of Egypt’s private universities [13], it is vital to evaluate students’ oral health behaviour in state-funded universities due to the palpable differences in the educational systems and circumstances between the private and public sectors of higher education in Egypt [9], which makes it inappropriate to generalise the results obtained from one sector to the other [13]. Aside from being almost 150 years older and better distributed nationwide compared to private universities, Egyptian state-funded medical and dental faculties embrace the majority of students in the medical field and commonly adopt different criteria and regulations for admission [9]. Furthermore, in recent years, several private universities have started to implement an integrated curriculum with problem-based learning, community-based education, and early clinical exposure, in contrast to the discipline-based curricula of most public dental faculties in Egypt [9]. In the current survey, Ain Shams University was chosen as a model for Egyptian state-funded universities. As reported in previous studies, its medical and dental faculties are considered among the oldest and most prominent in Egypt and its capital, Cairo. Aside from Cairo University, it encompasses the largest number of enrolled university students in Cairo and major Egyptian cities [9]. These students come from all of the different socioeconomic classes of Egyptian society, and there are even some that come from foreign countries.

In conformity with the generally reported distribution of the genders in dental education [28] and in Egyptian society [29], the population of the current study included more female (52.3%) than male participants (47.7%) (Table 3). Nearly 80% of the participating students lived with their families during their university studies, as described in previous investigations about students in Egypt [30] and other Arab countries [31], in contrast to European or Asian countries [3] (Item 1). Within the student population, females presented better oral health attributes than males, as stated previously in different countries [3,20,32,33,34]. This observation was equally remarked upon in relation to the general Egyptian population [6] and could be ascribed to the evidence that females frequently pay more attention to their appearance, general health, and body and demonstrate a better awareness regarding behaviour that improves their oral and dental health [3,34].

The general HU-DBI-based results of the current investigation exposed noticeably weak oral health attitudes and behaviour among most students of the study population in comparison to other dental students worldwide [3,21,35]. Almost one-third or more of both study groups never visited a dental practice (Item 2) or only visited the dentist in emergencies (Item 3). Yet, the higher rate of regular dental visits among clinical students displayed similar outcomes to a private university in Egypt [13], as well as Middle Eastern countries with similar oral hygiene behaviour, such as Turkey [12] and Saudi Arabia [14] (Items 2 and 3). Interestingly, dental student populations with better oral health outcomes in Germany and several European countries [3] conveyed contrasting results, with higher percentages of dental visits by preclinical students. This could possibly be explained by the potential for dental check-ups of clinical students within the scope of their clinical courses by other colleagues or clinical supervisors [3]. However, this possibility seems to be less conceivable or desired by students in Egypt or similar Middle Eastern societies, in which university- or public-based patient treatment is complementary and dedicated to the lower classes of society [36]. In agreement with earlier outcomes from the private educational sector in Egypt, as well as from other countries such as Jordan, Turkey, Saudi Arabia, Lithuania, India, and Germany [3,12,13,14,37], clinical students at Ain Shams University exhibited more frequent toothbrushing of every tooth (Item 9), at least twice a day or after every meal (Items 4 and 5), using a gingival health-favouring toothbrush and a professional brushing technique (Items 7 and 8). The stated outcomes of Items 4, 5, 7, 8, and 9 also conform with the observed statistically significant outcome for the preclinical students in terms of more pronounced gingivitis and unacceptable gingival aesthetics (Item 15), as well as frequent gingival bleeding after toothbrushing (Item 6), all of which clearly indicate the superior periodontal health of the clinical participants. Moreover, this could also be associated with the higher score for the regular use of mouthwash (Item 12) and scarcer dental flossing (Item 11) among the preclinical students of this survey. As well as reporting positive correlations between flossing and toothbrushing frequencies, previous studies have observed that mouthwash was used by higher percentages of individuals with gingival inflammation and lower levels of education [38]. However, obviously inconsistent with previous results [12,13,14,35] and similar to the results of preclinical students at German universities [3], the preclinical students at Ain Shams University appeared more observant in regard to their dental aesthetics and halitosis (Items 13 and 16) than their clinical colleagues, as well as appearing to care more about oral health-advantageous nutrition (Item 20). This reflects the motivation of preclinical students toward their own health, as observed in previous examinations [3,39], which often highlight the lack of knowledge and education necessary to correctly accomplish desirable oral health behaviour [3,13]. This knowledge deficiency was displayed in the current survey, as preclinical students believed it was possible to brush their teeth properly without toothpaste (Item 14), used the wrong type of bristles and brushing techniques (Items 7–9), and reported deteriorating teeth despite brushing (Item 10).

Smoking is a worldwide hazard to systemic and oral public health [3,40]. Oral healthcare professionals play a key role in encouraging their patients and society to cease smoking [41]. Egypt is one of 15 countries globally with a distressing burden of smoking-associated health problems, having an estimated 20.3% smoking prevalence among the general population [42]. In the current survey, the smoking prevalence was (31.0%) in preclinical and (28.2%) in clinical students (Item 17). More preclinical students reported smoking 10 or more cigarettes per day (18.3%) for a year or more (23.9%) in comparison to their counterparts (17.9% and 21.8%, respectively; see Items 18 and 19). This is an alarming result, as both study groups of dental students presented a smoking incidence higher than the general Egyptian population [42] and Egyptian medical students [43], and ranked among the highest rates worldwide [3,44]. In contrast to student groups in Japan [45], India [46], and Germany [3], the current survey displayed higher smoking rates among preclinical than clinical participants. This observation was explained in previous studies on medical and dental students, as higher levels of education or clinical knowledge were positively correlated with a cessation of tobacco-associated habits or the consumption of other psychoactive substances [47,48,49].

Observing all of the aspects of this survey and the HU-DBI mean score of both student groups, we found that the preclinical and clinical dental students at Ain Shams University exhibited overall weak attitudes and behaviour in relation to oral health when compared to similar groups in European, Asian, and Middle Eastern countries [3,12,21,35]. This reflects the previously described declining general status of public oral health in Egypt among different groups of the Egyptian population [8,25,26]. Nevertheless, the clinical participants of the current survey displayed a slightly better and statistically significant HU-DBI mean score than the preclinical students. This could be explained by the increasing clinical dental experience, with students finding themselves in regular contact with patients during their courses. As students develop in their dental education, they can become more conscious about their oral health and gain more dental knowledge that allows them to become more attentive of their patients and to instruct them appropriately [13]. This result conforms with various investigations performed in different countries [12,15,16,17,18,19,20] and in the Egyptian private educational health sector [13] and proposes a weak but noticeable effect in relation to students’ preclinical–clinical adaptation. Although this transition can be noticed in the current study, the overall poor oral health behaviour displayed could point to potential complications throughout different stages of the educational process [11]. One of the aspects that might potentiate the poor rates of oral health behaviour and attitudes detected in both study groups might be the increasing stress during dental studies, particularly during the last preclinical year and first clinical years, due to performance pressure, workload, social stressors, educational requirements, and interpersonal and environmental changes [3,50,51,52,53]. With higher stress levels, dental students show tendencies to disregard their oral health and correct dental behaviour [54]. This anxiety could even display stronger effects on participants living away from their families [55,56], females [57], and younger age groups [51]. These factors have more relevance to the preclinical group in the current survey, which displayed a reduced status of oral health compared to its counterpart group. Additionally, it is possible that the higher rate of smoking among the preclinical students is an element affecting the colour of their gingiva, as detected in Item 15 of the investigation [58], and could also be associated with increased academic stress, as mentioned above, which has previously shown significant effects on periodontal health due to immune modulations and endocrine changes [59,60,61].

Another essential element that should be considered as a major factor influencing oral health development in dental students is the content and structure of the university curricula [62,63,64]. In the last decade, most public medical and dental schools in Egypt have adopted a discipline-based curricular structure, depending mainly on large group lectures and classical methods of instruction and clinical exposure. These educational strategies are mainly oriented to the curriculum and rarely integrate the students involved [9]. This started to change recently when some Egyptian public and private universities introduced new modular parallel tracks, with an integrated curriculum featuring state-of-the-art educational methods, such as simulations, early exposure to clinical settings, communication skills training, and problem- and project-based learning. However, this development, which is still in the process of academic accreditation in many cases, has only been applied in a very small number of Egyptian universities to test the potential implications of this new system [9]. Regarding this curricular aspect, similar approaches might be needed in Ain Shams University and comparable state-funded Egyptian universities to accomplish the anticipated level of oral health progress throughout the entire process of dental education, specifically the preclinical–clinical student transition. As indicated by previous studies, the challenging shift from preclinical to clinical stages is a consequence of numerous and coinciding learning variables, including curricular content and design, as well as administrative difficulties, including the structure of classes or teaching methods applied in relation to large numbers of students or financial difficulties [11]. An early and ongoing exchange with clinical settings, in the form of an integrative education of preclinical and clinical teaching, can be proposed as an alternative solution to improve the results of the shifting intermediate stage between preclinical and clinical phases. Curricular evaluations and revisions to incorporate communication and social skills into dental educational programmes might assist students in becoming more patient-focused oral health experts [64]. This may also foster a practical understanding of the management of educational work-based anxiety and academic stress among students [64]. This curricular transition can subsequently prepare students to become responsible oral health educators, caring for the overall quality of life and wellbeing of their patients [11].

## 5. Limitations of the Study

The subsequent limitations of this study have to be recognised. One of the disadvantages of cross-sectional studies is the limited evaluation of data aside from the information gathered. However, similar studies have shown that this limitation of cross-sectional surveys has a weak effect on evaluation, as misdiagnoses of gathered data tend to be equally distributed [65]. In this survey, the detected oral health behaviour and attitudes of preclinical and clinical dental students at Ain Shams University cannot be completely ascribed to the examined aspects and the sociodemographic data. Investigating additional co-variables and elements such as the age, family income, and socioenvironmental settings of the students might display major effects on the survey results. It would surely be preferable if a sociodemographic examination could be added to the survey in line with an evaluation of the same participants in both their preclinical and clinical phases, with supplementary clinical assessment of their dental and gingival health.

Furthermore, as a pilot study, this survey only inspected a comparatively small sample of students from Ain Shams University to represent public university students in Egypt. The results attained might not be completely illustrative of other Egyptian public universities and cannot be generalised to all dental students in Egypt. However, this pilot study provides—for the first time—an important indication of the overall oral health attitudes and behaviour of students at state-funded Egyptian universities due to the relative similarity between most public universities in Egypt and their curricular and administrative differences when compared to most private educational health institutions, as mentioned above. Another noteworthy limitation of this exploration is the possible incidence of social desirability bias among the participants when answering the survey questions. Students might be inclined to give answers on the basis of textbook recommendations, which often do not mirror their real oral health behaviour. This tendency might have remained despite the fact that confidentiality was assured to participating students. Finally, the evaluation of simple items of the HU-DBI, such as the frequency of toothbrushing, does not provide a full representation of the overall oral health of students and could be reinforced in future studies by additional clinical assessments.

## 6. Conclusions

Clinical dental students at Ain Shams University in Egypt exhibited a slightly higher HU-DBI mean score for oral health attitudes and behaviour than preclinical students, with statistically significant differences. This reveals the moderately successful transition of the students from preclinical training to the clinical phase of education, where they will accomplish their function as instructors of oral health. However, both student groups showed an overall poor status in relation to oral health behaviour, highlighting the imperative need for significant changes in the educational system in public universities in Egypt. Curricular reassessment and restructuring are suggested to attain the anticipated level of oral health conduct among dental students and to support a completely successful preclinical–clinical transition. More investigations supplemented with clinical evaluations are recommended for the future to enable a meticulous inspection of the oral health status in dental students from different academic phases and for a more profound examination of the oral health attitudes and behaviour of Egyptian university students.

## Figures and Tables

**Table 1:** The modified English version of the Hiroshima University-Dental Behaviour Inventory (HU-DBI) survey in this study. Answers favouring correct oral health attitudes and behaviour are marked with (C).

Item 1	I live with my family now	Agree	Disagree
Item 2	I had been to a dentist office before	Agree (C)	Disagree
Item 3	I do not go to the dentist unless I feel pain	Agree	Disagree (C)
Item 4	I brush my teeth at least twice a day	Agree (C)	Disagree
Item 5	I brush my teeth after every meal	Agree (C)	Disagree
Item 6	My gums bleed when I brush my teeth	Agree	Disagree (C)
Item 7	I have been taught a professional brushing technique and I use it	Agree (C)	Disagree
Item 8	I use a toothbrush with hard bristles	Agree	Disagree (C)
Item 9	I brush each of my teeth carefully	Agree (C)	Disagree
Item10	I think my teeth are getting worse despite my daily brushing	Agree	Disagree (C)
Item 11	I use dental floss regularly	Agree (C)	Disagree
Item 12	I use a mouthwash regularly	Agree (C)	Disagree
Item 13	I worry about having bad breath	Agree (C)	Disagree
Item 14	I think I can clean my teeth without toothpaste	Agree	Disagree (C)
Item 15	I am bothered by the colour of my gums	Agree	Disagree (C)
Item 16	I worry about the colour of my teeth	Agree (C)	Disagree
Item 17	I am a smoker	Agree	Disagree (C)
Item 18	I smoke 10 or more cigarettes a day	Agree	Disagree (C)
Item 19	I have been smoking for a year or more	Agree	Disagree (C)
Item 20	I like snacking on sweets during the day	Agree	Disagree (C)

**Table 2:** Distribution of participating students by academic year and gender.

Academic Year (AY)	Total Number of Students (%)	Female Students (%)	Male Students (%)
1	25 (16.8)	13 (16.7)	12 (16.9)
2	24 (16.1)	13 (16.7)	11 (15.5)
3	22 (14.8)	13 (16.7)	9 (12.7)
4	19 (12.8)	7 (9.0)	12 (16.9)
5	21 (14.1)	11 (14.1)	10 (14.1)
6	38 (25.5)	21 (26.9)	17 (23.9)
Total number	149 (100)	78 (100)	71 (100)

**Table 3:** Distribution of participating students by preclinical or clinical study phases/academic years (AYs) and gender.

Study Phase	Total Number of Students (%)	Female Students (%)	Male Students (%)
Preclinical (AY 1–3)	71 (47.7)	39 (54.9)	32 (45.1)
Clinical (AY 3–6)	78 (52.3)	39 (50.0)	39 (50.0)
Total number	149 (100)	78 (52.3)	71 (47.7)

**Table 4:** Percentages and analysis of correct/incorrect (“agree” or “disagree”) responses to items pertaining to oral health attitudes (OHAs) and oral health behaviour (OHB) by preclinical and clinical dental students.

Item Number and “Keyword”	Number of Responses Favouring Correct Oral Health (%)		Number of Responses Contradicting Correct Oral Health (%)		*p*-Value
	Preclinical	Clinical	Preclinical	Clinical	
1 “Living with family” (sociodemographic) *(For statistical analysis, correct answers were matched with “agree” and incorrect answers with “disagree”)*	54 (76.1)	61 (78.2)	17 (23.9)	17 (21.8)	0.76
2 “Visits to the dentist” (OHA)	47 (66.2)	56 (71.8)	24 (33.8)	22 (28.2)	0.46
3 “Visiting the dentist only when in pain” (OHA)	32 (45.1)	41 (52.6)	39 (54.9)	37 (47.4)	0.36
4 “Toothbrushing twice a day” (OHB)	44 (62.0)	52 (66.7)	27 (38.0)	26 (33.3)	0.55
5 “Brushing after every meal” (OHB)	29 (40.8)	41 (52.6)	42 (59.2)	37 (47.4)	0.15
6 “Bleeding gums when brushing” (OHB)	40 (56.3)	50 (64.1)	31 (43.7)	28 (35.9)	0.33
7 “Professional brushing technique” (OHB)	40 (56.3)	49 (62.8)	31 (43.7)	29 (38.2)	0.42
8 “Toothbrush with hard bristles” (OHB)	37 (52.1)	51 (65.4)	34 (47.9)	27 (34.6)	0.10
9 “Brushing each tooth” (OHB)	40 (56.3)	50 (64.1)	31 (43.7)	28 (35.9)	0.34
10 “Teeth get worse despite brushing” (OHB)	32 (45.1)	39 (50.0)	39 (54.9)	39 (50.0)	0.55
11 “Regular dental floss” (OHB)	29 (40.8)	41 (52.6)	42 (59.2)	37 (47.4)	0.15
12 “Regular mouth wash” (OHB)	35 (49.3)	34 (43.6)	36 (50.7)	44 (56.4)	0.49
13 “Worrying about bad breath” (OHA)	53 (74.6)	57 (73.1)	18 (25.4)	21 (26.9)	0.83
14 “Tooth cleaning without toothpaste” (OHA)	36 (50.7)	40 (51.3)	35 (49.3)	38 (48.7)	0.94
15 “Bothered by the colour of gums” (OHB)	27 (38.0)	44 (56.4)	44 (62.0)	34 (43.6)	0.03
16 “Worrying about the colour of teeth” (OHA)	51 (71.8)	51 (65.4)	20 (28.2)	27 (34.6)	0.40
17 “Smoker” (OHB)	49 (69.0)	56 (71.8)	22 (31.0)	22 (28.2)	0.71
18 “10 or more cigarettes a day” (OHB)	58 (81.7)	64 (82.1)	13 (18.3)	14 (17.9)	0.96
19 “Smoking for a year or more” (OHB)	54 (76.1)	61 (78.2)	17 (23.9)	17 (21.8)	0.76
20 “Snacking on sweets during day” (OHB)	22 (31.0)	20 (25.6)	49 (69.0)	58 (74.4)	0.47

**Table 5:** HU-DBI scores of the preclinical/clinical female/male dental students (mean ± SD).

**HU-DBI score**	**Preclinical Students**	**Clinical Students**	***p*-Value**
	10.63 ± 2.64	11.50 ± 3.25	0.03
	**Female Students**	**Male Students**	***p*** **-Value**
	11.76 ± 2.79	10.35 ± 3.06	0.01

## Data Availability

The data presented in this study are available on request from the corresponding author.
